# Urinary T cells correlate with rate of renal function loss in autosomal dominant polycystic kidney disease

**DOI:** 10.14814/phy2.13951

**Published:** 2019-01-10

**Authors:** Kurt A. Zimmerman, Nancy M. Gonzalez, Phillip Chumley, Teresa Chacana, Laurie E. Harrington, Bradley K. Yoder, Michal Mrug

**Affiliations:** ^1^ Department of Cell, Developmental and Integrative Biology The University of Alabama at Birmingham Birmingham Alabama; ^2^ Department of Medicine The University of Alabama at Birmingham Birmingham Alabama; ^3^ Nephrology Research and Training Center The University of Alabama at Birmingham Birmingham Alabama; ^4^ Department of Veterans Affairs Medical Center Birmingham Alabama

**Keywords:** Adaptive immunity, cystic kidney disease, T cell subpopulations, eGFR, urine biomarker

## Abstract

Several innate immune response components were recognized as outcome predictors in autosomal dominant polycystic kidney disease (ADPKD) and their causative role in disease pathogenesis was confirmed in animal models. In contrast, the role of adaptive immunity in ADPKD remains relatively unexplored. Therefore, we evaluated T cell populations in kidney and urine of ADPKD patients using flow cytometry and confocal immunofluorescence microscopy approaches. These analyses revealed ADPKD‐associated overall increases in the number of intrarenal CD4 and CD8 T cells that were associated with a loss of polarity in distribution between the cortex and medulla (higher in medulla vs. cortex in controls). Also, the urinary T cell‐based index correlated moderately with renal function decline in a small cohort of ADPKD patients. Together, these data suggest that similar to innate immune responses, T cells participate in ADPKD pathogenesis. They also point to urinary T cells as a novel candidate marker of the disease activity in ADPKD.

## Introduction

Polycystic kidney diseases (PKD) are the most commonly inherited cause of end‐stage renal disease (ESRD). Autosomal dominant PKD (ADPKD; MIM: 173900 and 613095) leads to ESRD in most patients in their 50's while autosomal recessive PKD (ARPKD; MIM: 263200) is a major cause of ESRD in children. Currently, the majority of PKD patients can only be offered supportive care that unfortunately has minimal impact on the onset of ESRD (Spithoven et al. 2014; Fernando et al. 2017).

Innate immune system abnormalities are a dominant feature of both ADPKD (Zeier et al. 1992) and ARPKD (Mrug et al. 2013b). However, until recently, the PKD‐related innate immune response abnormalities were thought to be associated with advanced stages of disease progression without substantial impact on clinical outcomes. Discovery of abnormal urinary excretion of monocyte chemoattractant protein 1 (MCP‐1; also called chemokine (C‐C motif) ligand 2, CCL2) in early disease stages in ADPKD patients and animal models (Cowley et al. 2001; Zheng et al. 2003) changed this paradigm by demonstrating that innate immunity is altered early in the disease progression. Moreover, MCP‐1, as well as a monocyte/macrophage marker CD14, were identified as candidate predictors of ADPKD outcomes (Grantham et al. 2010; Zhou et al. 2010). Importantly, the addition of urine MCP‐1 data improved predictive models of renal function loss in ADPKD patients even when kidney volume, baseline kidney function, and demographic data were included in these models (Mrug et al. 2013a). In addition, several recent lines of evidence suggest that immune responses directly promote cystogenesis, at least in animal models (e.g., (Li et al. 2008; Mei et al. 2007; Shillingford et al. 2006)). Since the increase in macrophage markers precedes cyst formation in these models, the innate immune abnormalities may represent an early consequence of the underlying molecular PKD defect. Relevance of this concept was confirmed by studies showing that depletion of renal macrophages attenuated renal cystic disease progression in animal models (Karihaloo et al. 2011; Swenson‐Fields et al. 2013). Since, prominent mononuclear infiltrates were also observed in ARPKD (Zhou et al. 2010; Swenson‐Fields et al. 2013), the mononuclear cells likely participate in the disease pathogenesis through a common cystogenic pathway.

While mononuclear phagocytes including monocytes and macrophages represent central component of innate immune response, lymphocytes, another major renal immune cell type, are effector cells of adaptive immunity. Their subpopulations are engaged in complex, functionally relevant interactions with mononuclear phagocytes. Although renal interstitial lymphocytic infiltrates were described nearly three decades ago in patients with ADPKD (infiltrates of CD4 lymphocytes) (Zeier et al. 1992) and in renal cystic disease models (*Nek1* ^kat2J^ and *Nphp3* ^pcy^ mice and *Anks6* ^Cy^ (Han:SPRD Cy rats) (Kaspareit‐Rittinghausen et al. 1989; Takahashi et al. 1991; Vogler et al. 1999), systematic evaluation of lymphocytic subpopulations in ADPKD kidneys or their relevance in disease progression has not been reported. To advance this area of research, we characterized lymphocytic populations in kidneys from ADPKD patients. Also, inspired by recent description of urinary T cells as novel predictors of the disease activity in systemic lupus (SLE) (Enghard et al. 2009; Kopetschke et al. 2015), we adopted this method to analyze the association of urinary T cells with rate of decline in renal function in ADPKD patients.

## Materials and Methods

### Kidney tissue and urine samples

**Figure 1:**
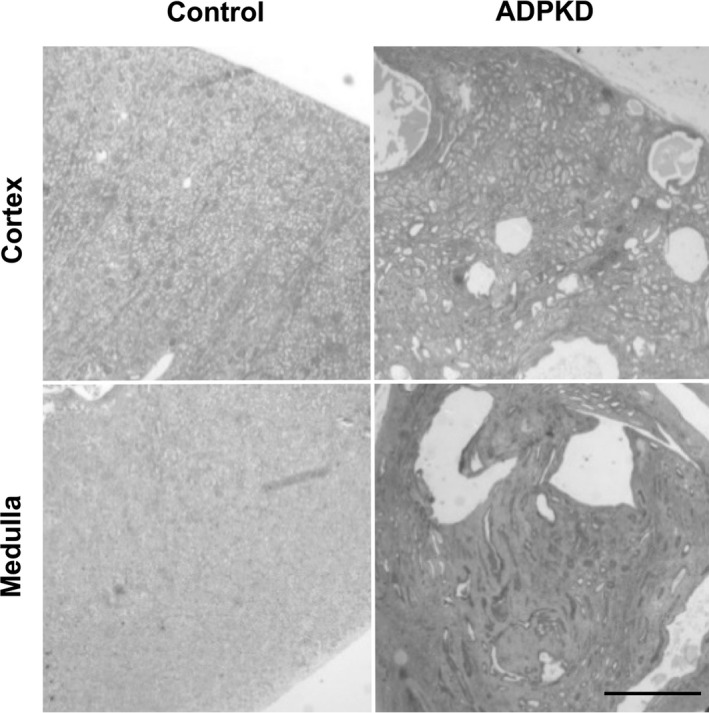
Examples of ADPKD and control renal tissues. Representative hematoxylin and eosin‐stained sections of tissues that were used in our study. These tissues contain relatively small amount of microscopic cysts and fibrosis because they were collected from normal‐appearing compartments of ADPKD kidneys. Scale bar = 1 cm.

Remnant human ADPKD and non‐PKD kidney tissues and urines were collected, de‐identified and analyzed according to a protocol approved by the Institutional Review Board of University of Alabama at Birmingham (UAB). All ADPKD tissues were obtained from patients with end‐stage renal disease (all pretransplant). All non‐PKD kidney tissues were collected from patients with intrarenal tumors; we collected normal‐appearing tissues from a kidney pole opposite to a well‐demarcated suspected malignancy; samples with large tumors were excluded. The glomerular filtration rate (eGFR) estimated by CKD‐EPI formula in non‐PKD controls was in the range of 54–107 (average 82) mL/min/1.73 m^2^ (Table 1). The average age of ADPKD patients was lower (52 ± 9 years vs. 64 ± 20 years for non‐PKD controls; *P* = 0.193). All tissues were processed after gross clinical pathology evaluation, within 4 h after removal from patients. Specifically, we collected representative samples of renal cortex and medulla; renal cortex was obtained from less than a 1 cm rim of tissue immediately under the renal capsule that contains glomeruli, renal medulla was collected from an approximately 2 cm rim of tissue without glomeruli adjacent to renal pelvis (Fig. 1). Validity of such an approach was confirmed by the presence of glomeruli in tissues assigned to correspond to cortex and the absence of glomeruli in tissues assigned to medulla. Tissues from immediate subcapsular and corticomedullary regions as well as renal papilla were excluded. In the case of ADPKD renal tissues, we collected only the most preserved, normal‐appearing parts of the kidneys with minimal amount of cysts or fibrosis present on gross evaluation (Fig. 1). Samples from patients with signs of systemic infection, renal infection, or renal/ureteral obstruction were excluded.

**Table 1:** Clinical characteristics of analyzed kidney tissues

	Control (*n* = 7)	ADPKD (*n* = 6)
Age [years]	64 ± 20	52 ± 9
Gender: male [%]	71	33
Race [%]		
African‐ American	29	66
Asian	0	14
Caucasian	71**	0
Hispanic	0	14
eGFR [mL/min/1.73 m^2^]	82 ± 24***	<10
Nephrectomy indication [%]		
Angiolipoma	14	–
Renal vessel injury	14	–
Renal cell carcinoma	71	–
Urothelial carcinoma	14	–

Control versus ADPKD: *P* < 0.01 (**); *P* < 0.001 (***).

The remnant urine samples were procured and analyzed within 2–4 h of collection using the same flow cytometry technique as used for kidney tissues. Relevant demographic, laboratory, and imaging data were recorded; results that were not obtained at the time of the urine collection (e.g., kidney size) were based on time‐adjusted earlier measurements. Samples from patients with suspected systemic, renal, or urinary tract infection based on history, exam, laboratory, or imaging studies (including urinalysis) were excluded.

### Flow cytometry of T cells from kidney tissues

The flow cytometry analyses were done on at least 3 g of renal cortex or medulla tissue (combined from multiple regions of the same kidney to reduce the selection bias). Each tissue was finely minced using a razor blade and incubated for 30 min in 5 mL of RPMI 1640 media (ThermoFisher Scientific) containing 1 mg/mL collagenase (Catalog#: C0130, Sigma‐Aldrich, St. Louis, MO) and 100 U/mL of DNase (Catalog#: D5025, Sigma‐Aldrich, St. Louis, MO). Single cells were obtained by passing digested tissue through a 70 *μ*mol/L filter (Catalog#: 352350, Corning, Corning, NY). Red blood cells were removed by lysis in ACK red blood cell lysis buffer (Catalog#: 118‐156‐101, Quality Biological, Gaithersburg, MD) and subsequent centrifugation at 220*g* for 5 min.

Labeling of cells for cytometry analyses was done according to manufacturers’ recommendations. Briefly, approximately 2 million cells were incubated in 1% BSA containing the following antibodies: eFluor 450 mouse anti‐human CD45 (Catalog#: 48‐9459‐42, 2D1; ThermoFisher Scientific (eBioscience), Waltham, MA), Brilliant Violet 605 mouse anti‐human CD3 (Catalog#: 317322, OKT3; BioLegend, San Diego, CA), PE mouse anti‐human CD4 (Catalog#: 317409, OKT4, BioLegend), APC‐Cy7 mouse anti‐human CD8 (Catalog#:557834, SK1, BD Biosciences, San Jose, CA), and aqua fluorescent reactive dye (L34957; ThermoFisher Scientific (Invitrogen)). Cells were washed with 1% BSA and resuspended in PBS.

All samples were analyzed on the LSRII flow cytometer (BD Biosciences) and the FlowJo version 10.0 software (FlowJo LLC, Ashland, OR).

### Flow cytometry of T cells from urine

The flow cytometry analyses were done from approximately 20–50 mL of remnant urine samples within 4 h of collection. Cells were isolated by centrifugation at 1200 rpm (220 g) for 10 min. Urine was discarded and the cells were washed with 1% BSA and respun at 1200 rpm for 5 min at 4°C. The isolated cells were stained for 30 min at room temperature with the antibodies (as outlined above in description of labeling of cells from kidney tissues). Cells were spun at 1200 rpm for 5 min at 4°C, washed with BSA, and fixed in 2% PFA for 30 min at room temperature. The labeled cells were resuspended in 1X PBS and analyzed using the LSRII flow cytometer.

### Immunofluorescence microscopy

The kidney tissue sections (7 *μ*m thick) were washed with 1X PBS, fixed with 4% PFA, and permeabilized with 1% Triton X‐100 for 10 min. These sections were incubated in PBS with 0.2% Triton X‐100 for 10 min followed by blocking with 1% BSA, 2% (vol/vol) donkey serum, and 0.02% sodium azide in PBS for 30 min at room temperature. Samples were then stained overnight at 4°C with the direct‐conjugated primary antibodies CD3‐PE (Catalog #: 12‐0034‐82, OKT3; ThermoFisher Scientific (Invitrogen)) and CD68‐Alexa647 (Catalog #:333819, Y1/82A, Biolegend) diluted in blocking solution. Slides were washed and stained with Hoechst (Sigma‐Aldrich; St. Louis, MO). Coverslips were mounted using Immuno Mount (ThermoFisher Scientific). Images were analyzed using a PerkinElmer ERS 6FE spinning disk confocal microscope (PerkinElmer, Inc., Waltham, MA).

### Statistical analyses

Statistical evaluations including outcome distributions and correlations were performed with SPSS 23.0 statistical software package (IBM Corp, Armonk, North Castle, NY.)

## Results

### T cell number is increased in ADPKD kidneys

**Figure 2:**
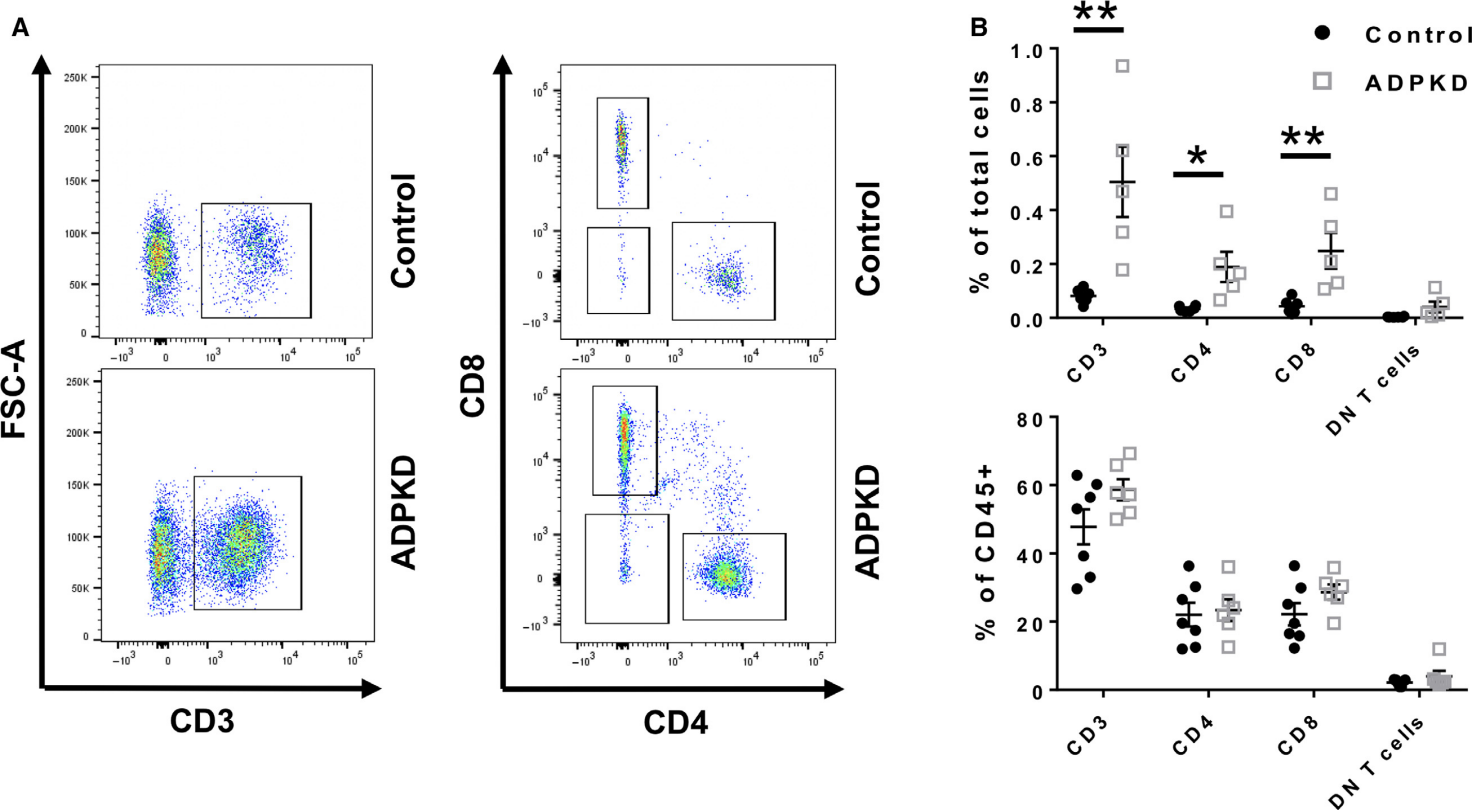
Total number of renal T cells are increased in ADPKD kidneys. (A) Representative flow cytometry plots showing CD3 T cells isolated from ADPKD and control kidneys (left panel) as well as CD4 and CD8 T cells (right panel). (B) Quantification of the total number of CD3, CD4, CD8, and DN T cells from normal (Black circles) and ADPKD kidneys (Gray squares) is shown as a percentage of total kidney cells and a percentage of CD45 immune cells (*P < 0.05, **P < 0.01). Each black circle (non‐ADPKD controls; *n* = 7) and each gray square (ADPKD patients; *n* = 6) represents a single patient.

The flow cytometry analyses of ADPKD (vs. non‐ADPKD) tissues revealed increased numbers of intrarenal CD3 T cells (0.500 vs. 0.081 percent of total renal cells; *P* = 0.006; Fig. 2) as well as their subtypes: CD4 T helper cells (0.189 vs. 0.034; *P* = 0.014), CD8 cytotoxic T cells (0.249 vs. 0.043; *P* = 0.008), and double negative (DN) T cells (0.041 vs. 0.004; *P* = 0.070). The ADPKD‐associated increase in T cells and their subtypes was proportional to the overall increase in intrarenal CD45 leukocytes in ADPKD (vs. non‐ADPKD) kidneys (Fig. 2).

**Figure 3:**
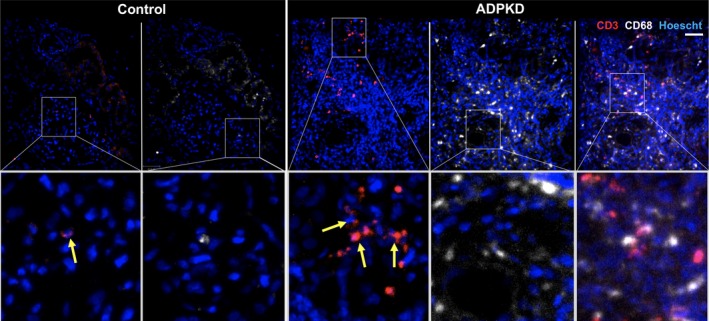
Interstitial T cell infiltrates are found in areas of small ADPKD cysts. Representative images demonstrate prominent CD3 T cell infiltrates as well as CD68 mononuclear phagocyte infiltrates in renal interstitium surrounding small, presumably developing, cysts. The control and ADPKD kidney sections were stained with antibodies to detect CD68 (white; a human pan‐macrophage marker) or CD3 (red, a pan T cell marker). Arrows depict individual T cells. Scale bar = 50 *μ*m.

Data obtained by immunofluorescence microscopy on kidney sections are consistent with that obtained by flow cytometry. Analysis of confocal images shows interstitial CD3 T cell accumulation in ADPKD (vs. non‐ADPKD kidneys), especially in regions containing smaller, presumably developing cysts. (Fig. 3). The increase of CD3 T‐cells as well as pattern of their distribution in ADPKD (vs. non‐ADPKD) kidney tissues resembled that of CD68, the human pan‐macrophage marker (Fig. 3) (Zeier et al. 1992; Swenson‐Fields et al. 2013).

### ADPKD abrogates polarity of T cell distribution in renal cortex versus medulla

**Figure 4:**
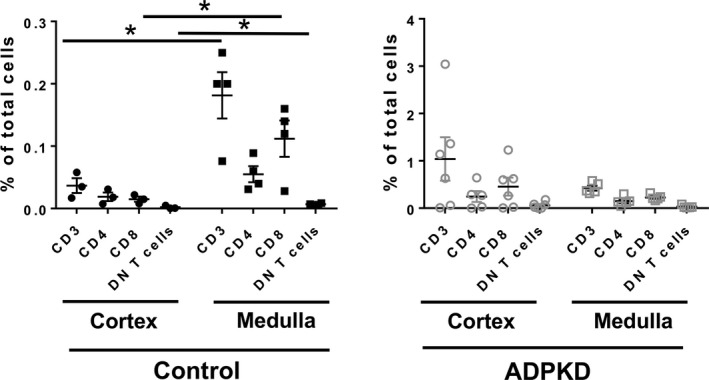
Influx of T cells to ADPKD kidneys abrogates normal polarity of T cell distribution in medulla versus cortex. The number of T cells and their subsets in the cortex versus medulla from control (left panel; cortex‐ black circles, medulla‐ black squares) and ADPKD kidneys (right panel; cortex‐gray circles; medulla‐gray squares;) is shown as a percentage of total kidney cells. *P < 0.05

**Figure 5:**
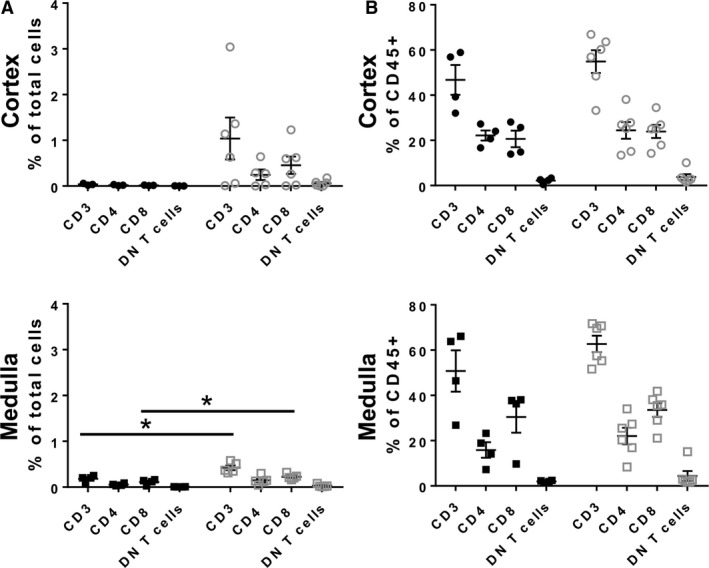
T cell accumulation is increased in renal medulla from ADPKD patients. (A) The numbers of T cells and their subsets in the cortex and medulla were determined by flow cytometry and are shown as a percentage of total kidney cells (black circles‐control cortex, gray circles‐ADPKD cortex; black squares‐ control medulla, gray squares‐ ADPKD medulla; *P < 0.05). (B) ADPKD had no effects on the proportion of T cells and their subsets as a percentage of CD45+ cells.

In normal human kidneys, flow cytometry analyses showed an increase in the number of CD3 T cells and their subtypes (CD4, CD8, and DN T cells) in renal medulla versus cortex (e.g., 0.182 vs. 0.037 for CD3 T cells, *P* = 0.023; 0.055 vs. 0.019 for CD4 T cells, *P* = 0.076; 0.112 vs. 0.015 for CD8 T cells, *P* = 0.038; 0.007 vs. 0.002 for DN T cells, *P* = 0.017; Fig. 4). This polarity was absent in ADPKD (i.e., 0.424 CD3 T cells in medulla vs. 1.039 for in cortex; *P* = 0.258) due to a suspected influx of T cells into both the cortex and medulla (Fig. 4). Overall numbers of CD3+ T cells and their subsets were increased in ADPKD kidneys compared to control kidneys although the number of T cells as a percentage of CD45+ cells was not different suggesting a general influx of immune cells in to the kidney (Fig. 5).

### T cells are present in urine of ADPKD patients and their numbers correlate with rate of renal function loss

**Figure 6:**
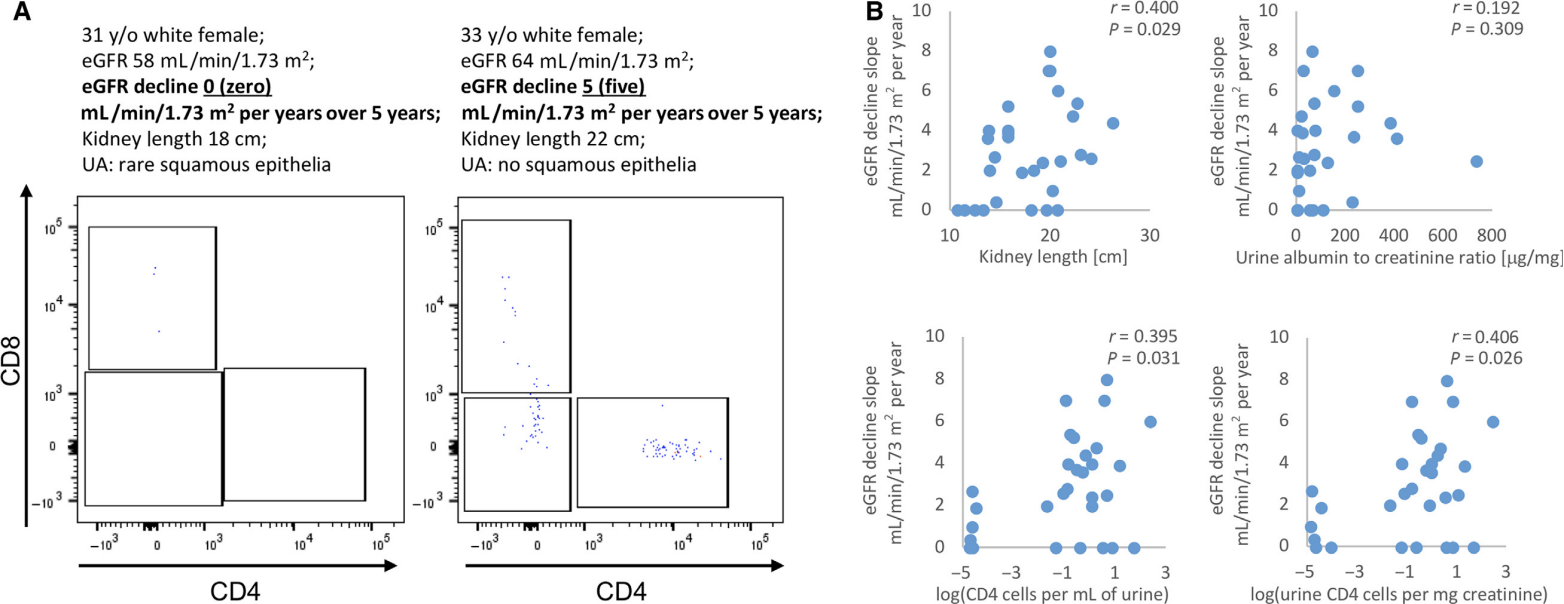
Urinary CD4 cells correlate with eGFR decline comparably but independently from size of ADPKD kidneys. (A) Representative flow cytometry plots of CD3 T cell subpopulations in ADPKD patients with similar demographic characteristics and kidney length, but a different rate of eGFR loss over time. The patient with eGFR decline of 5 mL/min/1.73 m^2^ per year over 5 years had moderate T cells numbers in the urine, while the patient with stable renal function (eGFR decline 0 over 5 years) had negligible amount of T cells in urine. (B) Correlation between the average yearly eGFR loss and urine CD4‐based indices (bottom panel) are comparable but independent to kidney length (upper left panel); both are superior to urine albumin to creatinine ratio (upper right panel).

Flow cytometry identified CD3 T cells in 97% (29 of 30) of analyzed urine samples from ADPKD patients with a range 0.04–360 cells/mL; on average the CD3 T cell subtypes were represented by CD4 (39%), CD8 (48%), and DN (16%) subsets. Evaluation of the associations between urine T cell counts and renal function indices showed moderately strong inverse correlation between eGFR and an index of CD4 cells (*r* = −0.455, *P* = 0.012). Importantly, there was also a moderately strong correlation between the urine CD4 T cell index and average annual rate of eGFR decline over 5 years (*r* = 0.411, *P* = 0.024; Fig. 6). Adjustment for urine volume instead of creatinine concentration yielded similar data.

Interestingly, the strength of correlation between kidney length (KL; recently identified predictor of ADPKD progression (Bhutani et al. 2015)) and the renal function indices showed similarly strong associations (e.g., for eGFR *r* = 0.380; *P* = 0.038; for the eGFR slope *r* = 0.400, *P* = 0.029). However, the T cell indices correlated only weakly with KL (e.g., *r* = 0.013–0.295) suggesting independent effects. Urine albumin to creatinine ratio, another predictor of ADPKD progression, correlated inversely with eGFR (*r* = −0.422; *P* = 0.201); however, its correlation with eGFR slope was weak (*r* = 0.192; *P* = 0.309).

## Discussion

Similar to the earlier report (Zeier et al. 1992), we observed increased interstitial T cell infiltrates in ADPKD (vs. non‐ADPKD) kidneys. However, we now provide a more granular quantitative assessment with flow cytometry‐based quantification of major T cell subtypes.

Our data complement existing studies that support a potential role of T cells in ADPKD pathogenesis. They include pericystic colocalization of T cells with CD68 mononuclear phagocytes (MPs), a group of cells that modulates renal cystic disease progression in animal models (Karihaloo et al. 2011; Swenson‐Fields et al. 2013). Functional multilevel interaction between T cells and MPs was well established; although, never before demonstrated in ADPKD. Since both T cells and MPs were more abundant in areas with smaller cysts (vs. larger cysts), it is possible that the interaction of T cells with MPs is important during initiation or early stages of the cyst formation rather than progressive growth of advanced larger cysts.

While the value of the reported flow cytometry analyses is limited by the relatively small size of ADPKD (*n* = 6) and non‐ADPKD cohorts (*n* = 7) that were not optimally matched by gender and race, the presented data are consistent with similar increases in intrarenal T cell subclasses in mouse cystic kidney disease models including the *Pkd1 p.R3277C (Pkd1* ^*RC/RC*^ *)* mouse that mimics human ADPKD phenotype (Kleczko et al. 2018).

The association of urine T cell indices with eGFR and eGFR slope reported in this manuscript provides additional support for the suggested role of T cells in ADPKD pathogenesis. Moreover, it points to urine T cells as a candidate marker of the disease activity in ADPKD that may complement structural ADPKD outcome predictors (e.g., as total kidney volume based indices). Urine T cells are already recognized as a biomarker for patients with proliferative lupus nephritis and used to monitor treatment response (Enghard et al. 2009; Kopetschke et al. 2015). Further studies in a larger patient cohort will be required to determine whether urinary T cells can be used as a marker of disease activity and response to therapy in ADPKD. Such biomarkers are sorely needed as the most sensitive approaches currently used to assess ADPKD progression are based on longitudinal follow‐up of total kidney volume (TKV). These data may require imaging many months to years apart to reveal a meaningful difference. The urinary T cell changes may occur more quickly, and their detection may allow for timely adjustments of future ADPKD therapeutics approaches.

The major limitation of this study is a relatively small size of an ADPKD cohort (*n* = 30) to evaluate association of urinary T cells with renal function indices. Also, this prospective cohort of consequent clinic patients does not meet the standard of well‐established ADPKD research cohorts that often include germline ADPKD mutations, as well as additional descriptors of the disease activity such as TKV.

Another limitation is the use of ADPKD kidneys from patients with end‐stage renal disease. These mostly fibrotic kidneys may not accurately reflect ADPKD pathobiology during earlier stages of the disease progression. However, the other potential sources of ADPKD kidney tissues are even more problematic: kidney biopsy is contraindicated in ADPKD and autopsy specimens are relatively rare, influenced by the underlying cause of death and collected over highly variable interval after the death. Instead, we suggest that urine is an important source of intrarenal T cells that can be obtained with minimal risk from ADPKD patients across different stages of the disease progression as we show in the current study.

In summary, we show that advanced ADPKD is associated with increased intrarenal CD4, CD8, and double negative (DN) T cells. Notably, ADPKD abrogated the normal polarity of intrarenal T cell distribution found in non‐ADPKD controls (low T cell content in renal cortex and high in medulla). While these data were based on tissues obtained from patients with end‐stage ADPKD, we observed nearly identical changes by flow cytometry of T cells from the urine of ADPKD patients. Also, the urinary T cell indices correlated moderately with renal function decline in a small cohort of ADPKD patients. Together, these data suggest that similar to innate immune responses, T cells participate in ADPKD pathogenesis. Also, they point to urinary T cells as a novel candidate marker of the disease activity in ADPKD.

## Conflict of Interest

M. M. reports grants and consulting fees outside the submitted work from Otsuka Pharmaceuticals and Sanofi.
